# Orbitotemporal Neurofibromatosis: Case Report

**DOI:** 10.1155/2012/498186

**Published:** 2012-03-29

**Authors:** Mahalakshmi Balasubramanyam, Goutham Cugati, Bipasha Mukherjee

**Affiliations:** ^1^Sankara Nethralaya, 18 College Road, Chennai 600034, India; ^2^ALNC, Post Graduate Institute of Neurological Surgery, VHS Hospital and Research Centre, TTTI Post, Taramani, Chennai 600113, India; ^3^Department of Orbit, Oculoplasty & Trauma, Sankara Nethralaya, 18 College Road, Chenna 600006, India

## Abstract

Plexiform neurofibromas occur in about 60% of neurofibromatosis type 1(NF-1) patients and can lead to severe morbidity by disfigurement or compression of vital structures. Moreover, these tumors can undergo malignant transformation. Both focal and localized bone abnormalities are part of the phenotypic expression of NF-1. We report a rare case of severe cranioorbital plexiform neurofibromatosis in a young male and discuss the classification, clinical features, and various treatment options of orbit-temporal neurofibromatosis type 1.

## 1. Introduction

 Neurofibromatosis type 1 or von Recklinghausen disease is an autosomal dominant genetic disorder, affecting approximately 1 in 3000 individuals [1]. Patients present with cutaneous plexiform neurofibromas which can undergo transformation to malignant peripheral nerve sheath tumors and rarely orbital abnormality.

## 2. Case Presentation

**Figure 1:**
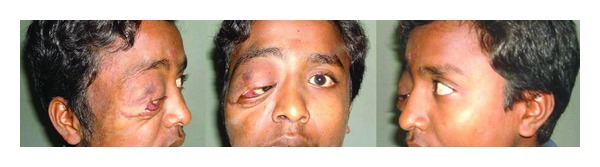
Clinical picture of the patient showing plexiform neurofibromatosis.

A 19-years-old Asian-Indian male presented in our oculoplasty department with complaints of painless progressive swelling around the right eye and right half of the face with protrusion of right eye since childhood. He gave history of surgical removal of the swelling elsewhere 10 years back followed by recurrence. External examination revealed severe ptosis in the right eye occluding visual axis and eccentric proptosis with the globe displaced inwards and medially. The lateral orbital rim was deformed with lower lid mechanical ectropion due to a soft tissue mass which had a “bag of worms” consistency on palpation (Figure 1). His vision was 1/60; N36 in the right and 6/6; N6 in the left eye. Right eye showed relative afferent pupillary defect and limited abduction. Slit lamp examination of the right eye showed temporal scleral thinning, clear cornea, and quiet anterior chamber with normal depth, partial posterior embryotoxon and clear lens. Lisch nodules were seen in both the eyes. Intraocular pressure with applanation tonometer was 18 and 12 mm of Hg in the right and left eyes, respectively.

**Figure 2:**
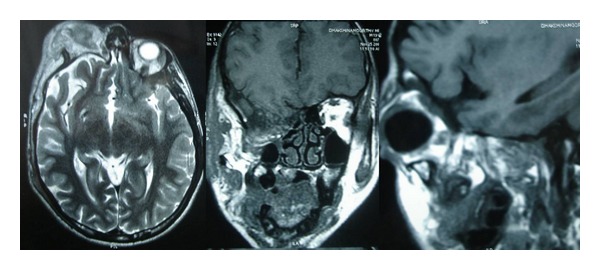
MRI of the brain axial, coronal, and sagittal views showing the absence of right sphenoid wing with herniation of the temporal lobe.

Fundus examination of right eye showed attached retina and normal macula with a tilted optic disc with cup disc ratio of 0.5 : 1 in both eyes. Left eye fundus was within normal limits. No other stigmata of neurofibromatosis were noted in the patient. MRI of the brain and orbits showed complete absence of the right greater wing, lesser wing, and half of the body of the sphenoid with herniation of the temporal lobe in the orbit (Figure 2). Multiple lobulated ill-defined soft tissue lesions were seen in the right forehead, temporal fossa, right preseptal soft tissue, cheek, infratemporal fossa, pterygopalatine fossa, and masticator space. The lesions were isointense in T1-weighted images and heterogeneously hyperintense signal in T2-weighted images. The clinical examination and imaging were diagnostic of plexiform neurofibromatosis. 

## 3. Discussion

Neurofibromatosis type I is one of the common single-gene disorders involving approximately 1 : 3000 individuals. The diagnosis of NF-I should meet at least two of the following criteria: (1) six or more café-au-lait spots (15 mm or larger after puberty and 5 mm or more in prepubertal individuals); (2) two or more neurofibromas of any type or one or more plexiform neurofibroma; (3) freckling in the axilla or groin; (4) optic glioma (tumor of optic pathway); (5) two or more Lisch nodules (benign iris hamartomas); (6) a distinctive bony lesion such as sphenoid wing dysplasia or thinning of the cortex of the bones; (7) a first-degree relative with NF-I [1]. 

Plexiform neurofibroma of the eyelid predominantly involves the upper lid which manifests as an S-shaped mechanical ptosis. Prominent corneal nerves are seen occasionally. Lisch nodules arise in the first decade and virtually all patients with NF-1 have them by 20 years. These nodules are melanocytic hamartomas, clear yellow to brown in color that appear as well defined, and smooth dome-shaped elevations projecting from the surface of the iris and often bilateral [2]. 

Less than 1% of patients suffering from NF-1 are reported to have abnormalities of the orbit [3]. Jackson et al. classified orbitotemporal neurofibromatosis into three groups [4]. First group had orbital soft-tissue involvement with a seeing eye. Krohel et al. advocated intact removal of a well-circumscribed lesion through an anterolateral orbitotomy [5]. Plexiform neurofibromas with diffuse soft tissue infiltration make complete resection difficult. Recurrent growth may lead to repeated surgeries and cosmetic deformity [6]. Surgical debulking can be done via an anterior, lateral, or anterolateral orbitotomy. Conservative blepharoptosis repair in patients with amblyopia may be sufficient. Second group had orbital soft-tissue and significant bony involvement with a seeing eye. In such patients an intracranial approach provides superior exposure and visualization for tumour debulking and posterolateral orbital wall reconstruction. This approach was first described by Marchac where a bilateral frontal bone flap is raised and the herniated temporal lobe is reduced into the middle-cranial fossa [7]. The lesion in the orbit is debulked. Opposite side frontal bone flap is split and the inner table is contoured to fill the defect of the roof and posterior wall of the orbit. Lateral orbital wall and floor are reconstructed. Surgical correction through an intracranial approach is associated with a higher complication rate. Friedrich et al. suggested the lateral orbital approach as an alternative technique, using intraoperative navigation system [8]. The use of computer-assisted simulation and navigation systems for the exploration of complex anatomic areas like the orbit has been reported to shorten overall operation time and to increase the safety of surgical maneuvers near delicate structures [9]. Third group had orbital soft-tissue and bony involvement with a blind, malpositioned eye. Treatment of such patients is limited to aesthetic procedures. Orbital exenteration is a viable option and often produces the best cosmetic results which may relieve the patient from major psychosocial distress due to facial disfigurement [3]. During surgery, the greater wing of sphenoid is reconstructed with split rib grafts. The superior and inferior orbital rims along with orbital floor are reconstructed and orbital prosthesis is placed at a later date. If the tumor is large and stable without progression, then observation may be a prudent option [8]. Our patient belonged to the third group who had orbital plexiform neurofibroma with sphenoid wing aplasia and a densely amblyopic eye. He was explained about the surgical options and the possible complications. He was not willing for any surgical intervention and hence was advised to follow up at regular intervals. Orbitotemporal neurofibromatosis being a rare entity with limited literature, management has been a challenging task for ophthalmologists, plastic surgeons, and neurosurgeons. With the advancement in technology, the management should depend on the clinical features, the extent of involvement, and the patient's expectations.
